# Evaluation of IL-4 and IL-13 Single Nucleotide Polymorphisms and Their Association With Childhood Asthma and Its Severity: A Hospital-Based Case-Control Study

**DOI:** 10.7759/cureus.57465

**Published:** 2024-04-02

**Authors:** Himamoni Deka, Mir A Siddique, Sultana J Ahmed, Pranabika Mahanta, Putul Mahanta

**Affiliations:** 1 Anatomy, Gauhati Medical College, Guwahati, IND; 2 Ophthalmology, Gauhati Medical College, Guwahati, IND; 3 Community Medicine, Assam Medical College, Dibrugarh, IND; 4 Obstetrics and Gynaecology, Jorhat Medical College, Jorhat, IND; 5 Forensic Medicine and Toxicology, Nalbari Medical College and Hospital, Nalbari, IND

**Keywords:** allele, gene, single-nucleotide polymorphism, childhood asthma, interleukin 13, interleukin 4, mutation

## Abstract

Background and objectives: Asthma is a common, chronic, atopic respiratory disease that is on the rise among children and adults worldwide. Various environmental, genetic, and biological interactions contribute to the surge in susceptibility to this disease. Interleukin (IL) genes, particularly IL-4 and IL-13, have been linked to asthma pathogenesis. The present study aims to investigate the genetic aberrations, specifically single nucleotide polymorphisms (SNPs) of IL-4 and IL-13, and their association with childhood asthma and its severity.

Methods: An unmatched hospital-based case-control study was conducted in a tertiary care hospital in Assam, India. The sample size was calculated to be 120 (60 cases and 60 controls) using the Epi Info software version 7.2 (Centers for Disease Control and Prevention, Atlanta, GA, USA), assuming a confidence interval of 95%, a power of the study at 80%, a ratio of control to cases as 1, a proportion of controls with exposure at 22%, and a proportion of cases with exposure at 46%. A total of 53 clinically diagnosed cases of childhood asthma in the age range of three to 12 years and 39 healthy controls free from respiratory diseases and having no history of asthma and/or allergy of the same age group attending a tertiary care hospital were included in the study. Children who never had asthma or allergies and who did not suffer from any upper or lower respiratory infections for the previous four weeks were considered controls. Prior informed consent and ethical clearance were obtained. Very seriously ill cases and controls were excluded from the study. The genetic investigation used polymerase chain reaction (PCR), followed by restriction fragment length polymorphism (RFLP), to discover SNPs in the IL-4 and IL-13 genes. Sequencing analysis was done for the cases with +2044 G>A of the IL-13 gene in relation to the severity of the disease. The difference in the proportions of specific SNPs between cases and controls was analyzed using the χ2^ ^test (a p-value of <0.05 was considered significant).

Results. Both the rs2070874 and rs2243250 polymorphisms of IL-4 showed no statistically significant associations. The mutation of the IL-13 gene in 1111C>T was higher among cases than controls. Both genotypic and allelic distributions of the +2044G>A polymorphism of the IL-13 gene revealed a significant association (p<0.05) with the severity of the disease.

Conclusion: Genetic aberrations in SNPs of IL-4 and IL-13 are prevalent among the pediatric patients of the study region. The SNP +2044G>A of IL-13 is instrumental in disease manifestation and severity among the pediatric population of the study region.

## Introduction

Asthma is a common, chronic, and rising atopic respiratory disease among children and adults worldwide. Atopic diseases, including asthma, eczema, and allergic rhinitis, have rapidly increased over the past two to three decades [1]. Lung inflammation, reversible airflow restriction, and increased airway responsiveness to various environmental stimuli are the main features of asthma. It is a phenotypically diverse condition with varying clinical manifestations [2]. Various studies have shown wide variability in the prevalence of atopic diseases among children in different parts of the world [3].

Both genetic predisposition and environmental triggers are attributed to the varying asthma rates among different communities. However, gene-by-environment interactions may potentially explain the global variation in asthma prevalence [4,5]. Various studies have revealed that asthma has a strong genetic association. Family studies and twin studies first established the genetic basis of asthma [6,7].

Global research over the past years suggests asthma is a complex genetic disease related to various genes involving several susceptible chromosomal regions [8]. Studies among different populations found that the cytokine gene clusters 5q31-33 contain asthma susceptibility genes [9]. Various studies have linked interleukin (IL) genes harbored in this gene cluster, particularly IL-13 and IL-4, to asthma pathogenesis [10]. Although there are observed ethnic differences, IL-4 polymorphisms have been linked to total IgE levels and may also be related to asthma and other allergy-related traits [11]. In human populations, IL-13 levels are increased in asthmatics chronically and in the lungs during attacks [12]. 

Although there is ongoing research worldwide with a focus in this direction, including many centers in India, literature is scarce in the north-east Indian population, especially on the contribution of genetic factors responsible for childhood asthma. Therefore, the present case-control study was undertaken at the Department of Anatomy and Paediatrics of a tertiary care hospital in Guwahati, AS, India, to identify genetic aberrations at the molecular level and their association with childhood asthma. The present work was carried out to investigate SNPs, in particular candidate genes IL-4 and IL-13, which may be responsible for disease causation and disease severity.

## Materials and methods

The present study is an unmatched hospital-based case-control study conducted in a tertiary care hospital in Guwahati, AS, India. The sample size was calculated to be 120 (60 cases and 60 controls) by using Epi Info software version 7.2 (Centers for Disease Control and Prevention, Atlanta, GA, USA), assuming a confidence interval of 95%, a power of the study set at 80%, a ratio of control to cases as 1, the proportion of controls with exposure at 22%, and the proportion of cases with exposure at 46% [13]. Clinically diagnosed cases of childhood asthma aged three to 12 years and controls of the same age group free from upper or lower respiratory infections, asthma, and/or allergies attending a tertiary care hospital in Guwahati between 2013 and 2017 were included in the study. Children who never had asthma or allergies and who did not suffer from any upper or lower respiratory infections for the four weeks prior to the study were considered controls. A total of 53 clinically diagnosed cases of childhood asthma and 39 healthy controls without asthma and/or allergy attending the same institution consented to participate in the study. Very seriously ill cases and controls were excluded from the study. Prior informed consent was obtained from parents of participants aged three to seven years, and informed consent of parents and assent of the children were obtained for children aged ≥ 7 years. If consent from parents and assent from children were not received, then those children were excluded from the study. 

The genetic investigation used the polymerase chain reaction (PCR)-restriction fragment length polymorphism (RFLP) method to discover SNPs in the IL-4 and IL-13 genes. Sequencing analysis was done for the cases with +2044 G>A of the IL-13 gene in relation to the severity of the disease. The infrastructure and facilities available in the Department of Anatomy, Department of Biotechnology (DBT) health care laboratory for human leukocyte antigen (HLA) tissue typing, and transplant immunology of the tertiary care hospital were utilized for the molecular genetics work.

Sample collection

**Figure 1:**
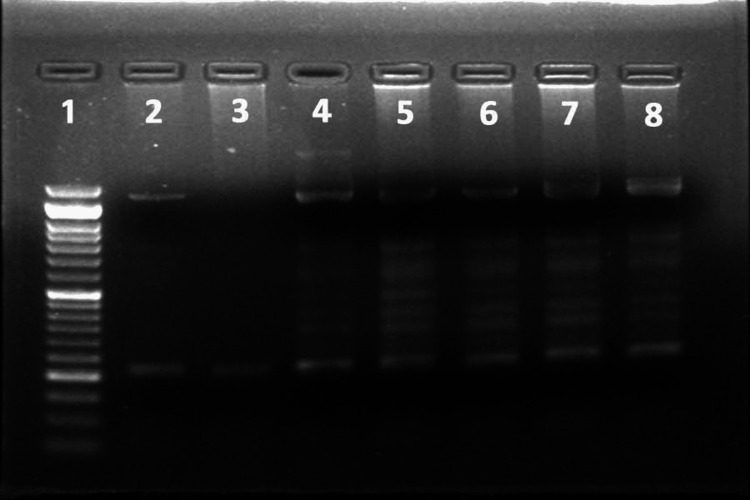
Standardization of PCR Lane 1 represents the 50 bp ladder. Lane 3 represents the PCR amplified product. PCR: Polymerase chain reaction

After obtaining informed consent, 2 ml to 5 ml of ethylenediaminetetraacetic acid (EDTA) blood under proper aseptic and antiseptic conditions was collected from each participant for genetic investigations. Standardization of techniques for DNA extraction and PCR was done (Figure 1). Standardization of PCR was done according to the manufacturer's instructions, and genomic DNA was isolated from peripheral blood leukocytes using the HiPura SPP Blood DNA Isolation Kit (Mumbai, MH, India). Nanodrop-quantified DNA was used for detecting the IL-4 and IL-13 gene SNPs, and the PCR-RFLP method was used as described elsewhere [14-16].

The purified PCR products were screened for sequencing changes by bidirectional sequencing. Sequencing was performed by Big Dye Terminator Mix version 3.1 (Applied Biosystems Inc., Foster City, CA, USA). All sequencing reactions were outsourced. ClustalW (BioEdit, Manchester, UK) was used for the analysis of files displayed using chromatogram files generated from the automated ABI Prism 3100 Genetic Analyzer (Applied Biosystems Inc.). Nucleotide sequences were compared with the published sequence of IL-13 (gene bank accession number chromosome 5, NC 00000.10 (132658173.132661109)) for the analysis of sequence changes, especially for the IL-13+2044 SNP, and the data obtained was statistically analyzed.

Genotype and allele frequency were analyzed in cases and controls. The IL-13+2044 SNP polymorphic genotype and allele frequencies were analyzed for disease severity. The institutional ethics committee of Gauhati Medical College (GMC) and Hospital (Guwahati, AS, India) approved the study (approval no. MC 233/2013/215).

Materials

The nanodrop (Thermofissure Scientific, Waltham, UK), incubator, vortex mixer, centrifuge (Thermofissure Scientific), and PCR (Thermofissure Scientific) were the equipment used in this study. The HLA tissue typing and transplant immunology laboratories in the Department of Anatomy at GMC are International Organization of Standardization (ISO)-in vitro diagnostics (IVD) certified, and the specifications meet standard guidelines. The reagents used were red cell lysis buffer (RCLB), white cell lysis buffer (WCLB), RNAase, elution (ET) buffer solution, 70% ethanol, isopropyl alcohol, and specific restriction enzymes for RFLP (Table 1).

**Table 1:** List of primers and restriction enzymes used for RFLP RFLP: Restriction fragment length polymorphism, PCR: Polymerase chain reaction

Primers (SNP)	Primer Sequence	Annealing temperature (ºC)	Restriction enzyme	PCR Product
rs2243250_F	TAAACTTGGGAGAACATGGT	50	AvaII	195 bp
rs2243250_R	TGGGGAAAGATAGAGTAATA			
rs2070874_ F	CAAGTTACTGACAATCTGGTGT	58	BsmA1	223bp
rs2070874_R	CGGCACATGCTAGCAGGAA			
−1111C>T_F	5-ACT TCT GGG AGT CAG AGC CA-3	60	Hpy991	372bp
−1111C>T_R	5-TAC AGC CAT GTC GCC TTT TCC TGC TCT TCC GTC-3			
4257G>A_F	5-CTT CCG TGA GGA CTG AAT GAG ACG GTC-3	60	NlaIV	236bp
4257G>A_R	5-GCA AAT AAT GAT GCT TTC GAA GTT TCA GTG GA-3			
+2044G/A_F	5'-CTT CCG TGA GGA CTG AAT GAG ACG GTC-3'	57	NlaIV	210bp
+2044G/A_R	5'-GCA AAT AAT GAT GCT TTC GAA GTT TCA GTG GA-3'			

Statistical analysis

Frequency distributions of different variables were observed between cases and controls. The allele frequencies of an individual allele for a specific SNP were calculated. The difference in the proportions of specific SNPs between cases and controls was analyzed using χ2 and unadjusted logistic regression. In addition, the particular SNPs found to have a genotypic or allelic association with the disease were further analyzed concerning disease severity using the χ2 test. Disease severity was categorized as per Global Initiative for Asthma (GINA) guidelines for the description of participants in epidemiological studies and clinical trials based on the prescribed treatment step. Patients who received step 2 treatment were classified as having mild asthma, steps 3 and 4 treatment as having moderate asthma, and step 5 treatment as severe asthma [17]. A p-value of <0.05 was considered statistically significant.

## Results

**Figure 2:**
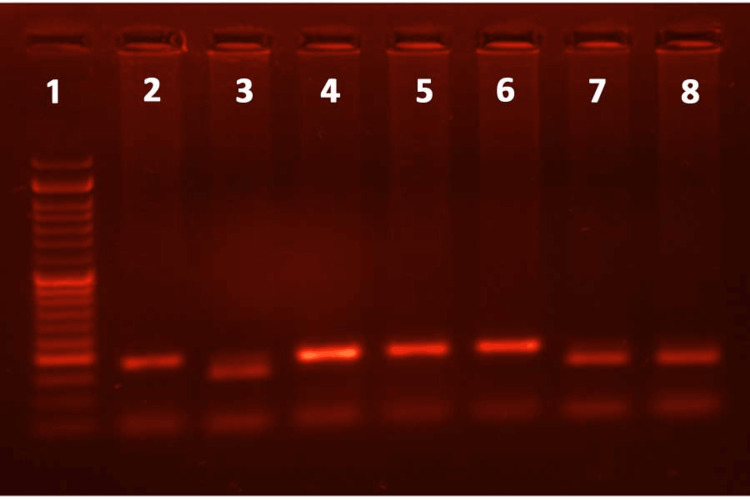
The PCR-RFLP gel electrophoresis for SNP C-33C>T of the IL-4 gene reveals an RFLP pattern obtained following digestion with restriction enzyme BsmA1. Lane 1 represents the 50 bp ladder; lanes 2, 4, 5, and 6 represent the typical RFLP pattern (wild type); lanes 3 and 7 represent the RFLP pattern in heterozygous mutant allele; and lane 8 represents a typical RFLP Pattern in homozygous mutant allele. PCR: Polymerase chain reaction, RFLP: Restriction fragment length polymorphism, SNP: Single nucleotide polymorphism

**Figure 3:**
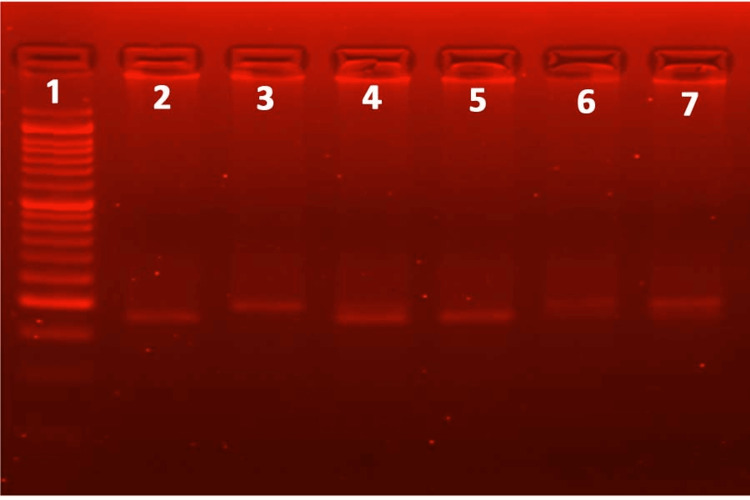
The PCR-RFLP gel electrophoresis for SNP C-589C>T of the IL-4 gene reveals the RFLP pattern obtained following digestion with the restriction enzyme AVAII. Lane 1 represents the 50 bp ladder; lanes 2 and 5 represent a typical RFLP pattern (wild type); lanes 4, 6, and 7 represent the RFLP pattern in the heterozygous mutant allele; and lane 3 represents the typical RFLP pattern in the homozygous mutant allele. PCR: Polymerase chain reaction, RFLP: Restriction fragment length polymorphism, SNP: Single nucleotide polymorphism

The genotypic distribution of the rs2070874 polymorphism of the IL-4 gene revealed a non-significant association with childhood asthma among the study participants (Figure 2). Furthermore, in the case of the rs2243250 polymorphism of the IL-4 gene, the genotypic distribution of mutant alleles was higher among cases (Figure 3). However, the association was not found to be statistically significant (Table 2).

**Table 2:** Genotype distribution for various polymorphisms of the IL-4 gene in cases and controls

Polymorphism	Genotype	Controls	Cases	OR (95% CI)	p-value
rs2070874	Wild (CC)	27 (31.76%)	45 (52.94%)	1	
	Heterozygous mutant allele (CT)	5 (5.88%)	5 (5.88%)	0.60 (0.16-2.26)	0.45
	Homozygous mutant allele (TT)	1 (1.18%)	2 (2.35%)	1.20 (0.10-13.87)	0.88
rs2243250	Wild (CC)	8 (9.30%)	13 (15.12%)	1	
	Heterozygous mutant allele (CT)	22 (25.58%)	31 (36.05%)	0.87 (0.31-2.44)	0.79
	Homozygous mutant allele (TT)	3 (3.49%)	9 (10.47%)	1.85 (0.39-8.92)	0.45

The frequency of wild allele C is higher in both cases and controls compared to the T allele for the rs2070874 polymorphism of the IL-4 gene. However, no significant association was found between the allele frequency and childhood asthma. In the case of the rs2243250 polymorphism of the IL-4 gene, individuals with the T allele had 1.17 times higher odds of childhood asthma than those with the C allele. Still, the association was not found to be statistically significant (Table 3).

**Table 3:** Distribution of genotype allele frequency for various polymorphisms of the IL-4 gene

Polymorphism	Allele type	Cases	Controls	OR	p-value
rs2070874	C	95	59	1 (ref)	0.67
	T	9	7	0.80 (0.28-2.26)	
rs2243250	C	57	38	1 (ref)	0.63
	T	49	28	1.17 (0.63-2.17)	

**Figure 4:**
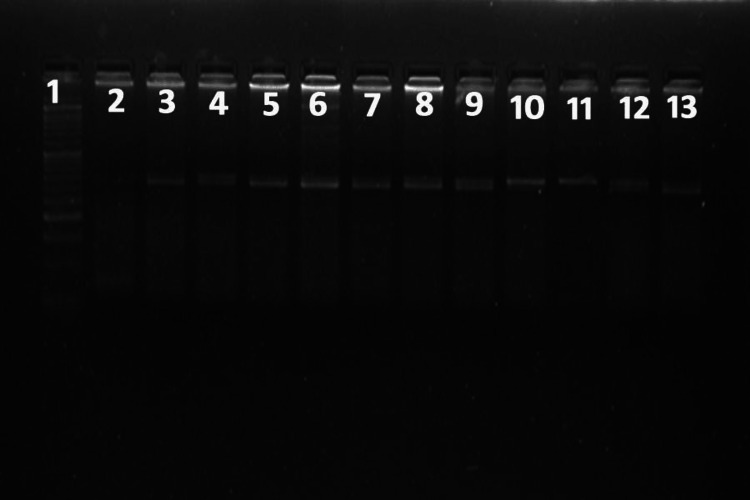
The PCR-RFLP gel electrophoresis for SNP -1111C>T of the IL-13 gene revealed an RFLP pattern obtained following digestion with the restriction enzyme HPY99I. Lane 1 represents the 50 bp ladder; lanes 3 and 5 represent the typical RFLP pattern (wild type); lanes 4, 6, 7, 8, 9, 12, and 13 represent a typical RFLP pattern in the heterozygous mutant allele; and lanes 10 and 11 represent the typical RFLP pattern in the homozygous mutant allele. PCR: Polymerase chain reaction, RFLP: Restriction fragment length polymorphism, SNP: Single nucleotide polymorphism

**Figure 5:**
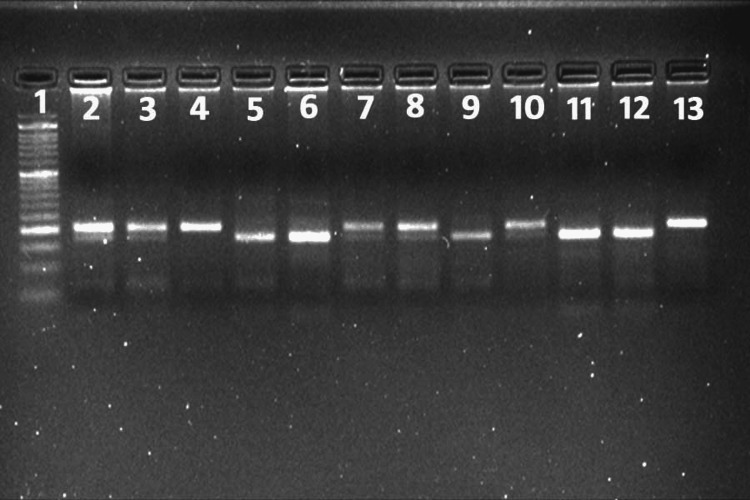
The PCR-RFLP gel electrophoresis for SNP 4257G>A of the IL-13 gene reveals an RFLP pattern obtained following digestion with the restriction enzyme NlaIV. Lane 1 represents the 50 bp ladder; lanes 5, 6, 9, 11, and 12 represent the typical RFLP pattern (wild type); lanes 2, 3, 7, 8, and 10 represent the typical RFLP pattern in a heterozygous mutant allele; lanes 4 and 13 represent a typical RFLP pattern in a homozygous mutant allele. PCR: Polymerase chain reaction, RFLP: Restriction fragment length polymorphism, SNP: Single nucleotide polymorphism

**Figure 6:**
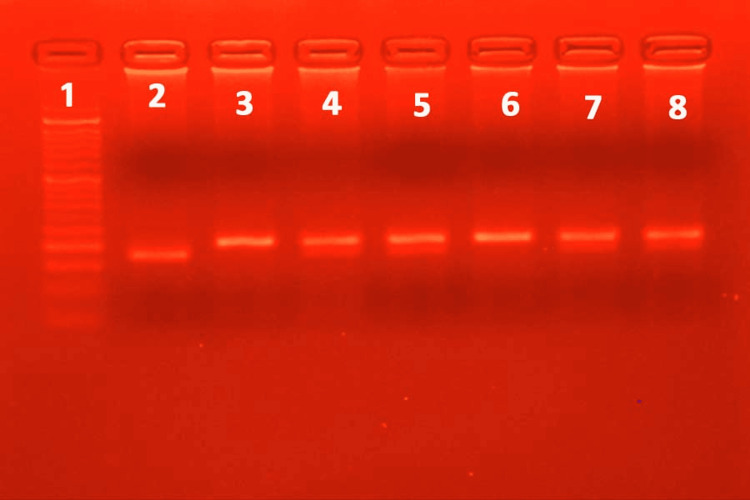
The PCR-RFLP gel electrophoresis for SNP +2044G>A of the IL-13 gene reveals an RFLP pattern obtained following digestion with the restriction enzyme NlaIV. Lane 1 represents the 50 bp ladder; lane 2 represents the typical RFLP pattern (wild type); lanes 4, 5, 7, and 8 represent a typical RFLP pattern in a heterozygous mutant allele; and lanes 3 and 6 represent the typical RFLP pattern in a homozygous mutant allele. PCR: Polymerase chain reaction, RFLP: Restriction fragment length polymorphism, SNP: Single nucleotide polymorphism

The mutation of the IL-13 gene in -1111C>T was higher among cases than controls (Figure 4). Patients with homozygous or heterozygous mutations of the IL-13 gene for -1111C>T polymorphism were found to have more than two times higher odds of developing the disease. However, the association was not statistically significant. In the cases of 4257G>A (Figure 5) and +2044G>A polymorphisms of IL-13 (Figure 6), no significant association was observed between genotype frequency and the disease (Table 4).

**Table 4:** Genotype distribution for various polymorphisms of the IL-13 gene in cases and controls

Polymorphism	Genotype	Controls	Cases	OR (95% CI)	p-value
-1111C>T	Wild (CC)	16 (20.00%)	11 (13.75%)	1	
	Heterozygous mutant allele (CT)	17 (21.25%)	25 (31.25%)	2.14 (0.80-5.72)	0.13
	Homozygous mutant allele (TT)	3 (3.75%)	8 (10.00%)	3.88 (0.39-8.92)	0.08
4257 G>A	Wild (GG)	8 (9.52%)	11 (13.10%)	1	
	Heterozygous mutant allele (GA)	10 (11.90%)	8 (9.52%)	0.58 (0.16-2.14)	0.41
	Homozygous mutant allele (AA)	21 (25.00%)	26 (30.95%)	1.20 (0.20-1.72)	0.97
+2044G>A	Wild (GG)	36 (43.90%)	24 (29.27%)	1	
	Heterozygous mutant allele (GA)	1 (1.22%)	12 (14.63%)	1.20 (0.10-14.69)	0.89
	Homozygous mutant allele (AA)	1 (1.22%)	8 (9.76%)	0.80 (0.06-10.11)	0.86

In the case of the IL-13 gene, the frequency of the T allele was higher among cases than controls in the case of the -1111C>T polymorphism. Although not statistically significant, the T allele was found to have almost two times higher odds of childhood asthma. For the +2044G>A polymorphism of IL-13, A allele frequencies were significantly higher among cases (p<0.05), with almost 11 times higher odds of disease (Table 5).

**Table 5:** Distribution of genotype allele frequency for various polymorphisms of the IL-13 gene

Polymorphism	Allele type	Cases	Controls	OR (95% CI)	p-value
-1111C>T	C	47	49	1( ref)	0.06
	T	41	23	1.86 (0.97-3.56)	
4257 G>A	G	48	37	1 (ref)	0.47
	A	42	41	0.79 (0.43-1.45)	
+2044G>A	G	60	73	1 (ref)	0.001
	A	28	3	11.36 (3.29-39.19)	

Concerning the +2044G>A polymorphism of the IL-13 gene, both genotypic and allelic distribution revealed a significant association (p<0.05) with the severity of the disease. Homozygous mutations were observed in most of the severe cases. Additionally, the allelic distribution showed a dominance of the A allele among the severe childhood asthma cases (Table 6).

**Table 6:** Genotype and allele frequency for +2044G>A polymorphism of IL-13 gene and its relationship with severity of childhood asthma

Polymorphic genotype	Severity of disease			p-value
	Mild	Moderate	Severe	
GG (n=24)	12 (50.0%)	11 (45.8%)	1 (4.2%)	< .001
GA (n=12)	4 (33.3%)	5 (41.7%)	3 (25.0%)	
AA (n=8)	0	0	8 (100%)	
Polymorphic allele				
G (n=60)	28 (46.7%)	27 (45.0%)	5 (8.3%)	<0.001
A (n=28)	4 (14.3%)	5 (17.9%)	19 (67.9%)	

## Discussion

Asthma and atopic disorders have been on the rise over the past few decades, involving both children and adult populations. Environmental factors, underlying biological factors, and inherited vulnerabilities cause asthma. Recent studies have suggested that genetic polymorphisms in the genes for immune pathways like IL-4 and IL-13 may be linked to the disease phenotype. The current study also investigates SNPs in IL-4 and IL-13, which may be responsible for disease causation and disease severity among pediatric patients in the northeastern region of India.

In the present study, with a higher wild genotypic frequency among cases, no significant association of SNP at position -33CT (rs2070874) of IL-4 was found with the disease. The observations are consistent with universal observations, except for studies in Iran and China [18-23]. While, in the case of genotype frequency for the rs 2243250 polymorphism of IL-4, maximum cases were seen with the heterozygous mutant allele (CT), the homozygous mutant allele (TT) is 1.85 times more risky for disease association than wild-type (CC). However, the association was not found to be statistically significant. Inducing heavy chain isotype switching, IgE synthesis by B-cells, and acting as a growth factor for Th2 cells, the cytokine IL-4 plays a vital role in the etiology and progression of allergic inflammation and atopy [24]. Several studies worldwide reported contradictory observations regarding SNP -589/C-590 T's association with asthma [8,10,18,25,26].

In the case of SNP-1111C>T of IL-13, the maximum cases were found with the heterozygous mutation (CT). The homozygous mutant allele (TT) was found to increase the risk of the disease by almost fourfold, although the association was not statistically significant. The findings are in concordance with similar studies [22,27,28]. However, in contrast to our findings, studies from Malaysia and China reported a highly significant association of the SNP-1111C>T of IL-13 with asthma [15,23]. Moreover, the homozygous mutant allele (AA) was higher among cases for the 4257GA polymorphism of IL-13 in the present study. However, the association was not found to be significant. Another study also reported a similar type of observation for SNP 4257GA [15]. Various studies have reported IL-13 as a crucial cytokine in the chronic inflammation of the airways. Asthma therapy experts have identified IL-13 as a potential therapeutic target since it plays a role in Th2 inflammation [28].

In the present study, the genotype distribution of the +2044GA polymorphism of IL-13 showed a statistically significant association with childhood asthma (pooled p-value <0.001). Furthermore, the A allele frequencies were significantly higher among cases, with almost 11 times higher odds of the disease. Our observations agree with similar studies [16,23].

Both genotypic and allelic distribution revealed a significant association (p<0.05) of SNP 2044G>A IL-13 with the severity of the disease in the current study. A preponderance of homozygous mutations in SNP 2044G>A was observed among the severe cases. Moreover, the allelic distribution showed a dominance of the allele A among the severe childhood asthma cases, which was statistically highly significant. These findings agree with another study [16].

As a premier tertiary referral center in the region, most patients coming to this health and research institute belong to India's entire northeastern region. Childhood asthma is a commonly encountered problem in the pediatric department of our hospital. It is especially concerning considering the genetic factors for disease association in this region. Genetic factors help identify people at risk of developing asthma. Proper preventive strategies for these high-risk patients will help reduce disease occurrence and decrease the precipitation of acute attacks.

Limitations

Sequencing is the gold standard for discovering the SNP, but it is quite an expensive investigation. Sequencing was done only for SNP +2044GA in selected samples in the present study. The easily affordable availability of sequencing techniques in an in-house environment can lead to a more efficient diagnosis of the disease at the genetic level. More research with many patients is required to determine the population-attributable risk for a specific SNP. The PCR-RFLP can be used to get an immediate preliminary diagnosis of asthma at the genetic level. Following it up with sequencing would give rise to a better general overview of disease etiology at the genetic level.

## Conclusions

Genetic aberrations in SNPs of IL-4 and IL-13 are prevalent among the pediatric patients of the study region. The SNP +2044G>A of IL-13 is instrumental in disease manifestation and severity among the pediatric population of the study region. The present study findings suggest that genetic factors play an essential role in childhood asthma and its severity. More in-depth research concerning this aspect in this region of the country will aid in providing a better overview of disease etiology at the genetic level. Understanding the clear etiology will help clinicians plan specific treatment protocols for pediatric patients suffering from asthma.
